# Mercy Hospital

**Published:** 1878-07

**Authors:** F. E. Waxham

**Affiliations:** House Surgeon, Mercy Hospital


### Clinical Reports.

### MERCY HOSPITAL.

*Aneurism of the Femoral Artery Cured by Compression.*—Reported by F. E. Waxham, M. D., House Surgeon, Mercy Hospital.

Antonio Corvini, Italian, aged 61, entered Dr. Andrews' surgical ward, March 17th, 1878, suffering from a very large aneurism of the femoral artery. Twenty days previous to entering the hospital he had noticed a pulsation in the lower third of the femoral artery, which appeared after a hard day's work. This pulsation increased rapidly in force, forming a tumor as large as a goose egg, and entirely disabling him. Upon entering the hospital, March 14th, the recumbent position was ordered, and the artery compressor applied just above the tumor. Pressure was exerted sufficient to arrest the pulsations in the aneurism, but it produced so much discomfort as to necessitate its removal, or a change of position, in a few moments. March 17th. Applied the artery compressor at several points above the aneurism, a few moments at a time.

March 19th. Finding the above means inefficient, compression was now made by two strips of board, each 10x3½ inches, placed across the thigh, one above and the other below. The under side being protected by a tin splint, well padded, the strips were fastened on the inner side by a bandage, and the free ends approximated by the artery compressor used as a clamp. This arrested the pulsations in the tumor, and was borne very well by the patient a few moments at a time.

March 20th. Still contiued the use of the clamp, but the aneurism seemed hardly less in size or force of pulsations. March

23d. The tumor seemed to be diminising slightly in size and increasing in firmness. March 24th. Patient kept up the pressure faithfully. March 26th. The walls of the aneurism seemed a little firmer. March 28th. Patient was put upon tr. aconiti and tr. veratri, to lessen the force and frequency of the heart's action.

April 3d. Pulse ranged from 52 to 56 per minute, from day to day. Pressure applied as usual during his waking hours. April 5th. No change was noted in the appearance of the tumor. April 12th. As the aneurism showed no further tendency to diminish in size, more vigorous measures were resolved upon, and continuous compression attempted. At 5 o'clock p. m., morphia gr. $\frac{1}{3}$ was given and repeated every hour and a half during the night. He bore the pressure very well for half an hour, and then became almost frantic with pain, crying like a child. When he could stand it no longer the position of the pressure was changed. Ten o'clock p. m. He bore the pressure better, as a result of the effects of the morphia. The aneurism seemed to be growing smaller and harder. Pulsations were now seen distinctly just above the knee in one of the anastomotic arteries. Eleven o'clock. Was sleeping. Compression had just been maintained for forty minutes without a change of position; the longest period to-night as yet. The aneurism was decidedly smaller, but the pulsations in the anastomotic branch seemed to be increasing. These pulsations were irregular, about thirty per minute, but quite forcible, so much so as to be distinctly heard when the ear was applied, and plainly detected by the hand, this seeming to indicate the formation of another aneurism. The patient said he felt as if snakes were crawling through his flesh below the compression; doubtless due to the blood forcing its way through the collateral circulation, and the consequent stretching of the vessels. Twelve o'clock p. m. Pulse at the wrist, 48 per minute and regular. At one a. m. continues the same. Three a. m. Left him in charge of the nurse. The pressure had to be shifted every half hour.

April 13th.—At 9 o'clock a. m. vomited after taking his morphia. No more medicine given during the day, but compression continued, shifting the position every few minutes as the pain necessitates; 5 o'clock p. m., gave morphia gr. $\frac{1}{3}$; at 7 p. m., morphia gr. $\frac{1}{2}$, causing nausea; at 9 p. m., gr. $\frac{1}{3}$, causing emesis. Morphia not repeated but pressure still continued during the night. The pulsation in the collateral circulation had ceased.

April 14th.—Tumor appeared harder and smaller. Pressure has been applied all day, but was omitted to-night in order to give him rest and sleep. Bowels moved four times to-day.

April 15th.—The aneurism appeared very much smaller. Compression had been applied all day, but no morphia used, the patient enduring the pressure about 15 to 20 minutes without shifting; 5 o'clock p. m. gave morphia gr. $\frac{1}{3}$, to be repeated every hour and a half, and pressure continued.

April 16th.—Tumor very much smaller.

April 17th.—Pressure not applied last night but continued during the day. Morphia given in $\frac{1}{3}$ gr. doses and pressure continued to-night.

April 18th.—Pulsations in the tumor had almost ceased.

April 20th.—Applied the pressure continuously for one hour to-day without a change of position, after which the pulsations were almost imperceptible.

April 23d.—Pressure applied during the day.

April 25th.—Pressure was applied several hours without intermission or a change of position, when the limb was found greatly swollen and œdematous, pitting upon pressure. Patient complained of great prostration, dimness of vision and distress at the stomach. Pressure was immediately removed and a little wine given.

April 26th.—Has been feeling very miserable to-day.

April 27th.—Was better; aneurism almost gone.

April 28th.—Pulsation in the tumor had entirely ceased.

April 29th.—Pressure applied to-day at patient's request, although the pulsations have not returned.

April 30th.—Was dressed and walked about the ward to-day.

May 1st.—Tumor quite hard and pulsations absent.

May 2d.—Left the hospital to-day entirely cured. Time in hospital 49 days.

The patient was seen May 27th. The tumor was small and hard, giving no inconvenience, and showing no disposition to return.

